# Targeting HDAC Complexes in Asthma and COPD

**DOI:** 10.3390/epigenomes3030019

**Published:** 2019-09-07

**Authors:** Martijn R. H. Zwinderman, Sander de Weerd, Frank J. Dekker

**Affiliations:** Department of Chemical and Pharmaceutical Biology, Groningen Research Institute of Pharmacy (GRIP), University of Groningen, 9713 AV Groningen, The Netherlands (M.R.H.Z.) (S.d.W.)

**Keywords:** asthma, COPD, inflammation, NF-κB, HDAC, acetylation, post translational modification (PTM), inhibitor, drug design, co-repressor complex, review

## Abstract

Around three million patients die due to airway inflammatory diseases each year. The most notable of these diseases are asthma and chronic obstructive pulmonary disease (COPD). Therefore, new therapies are urgently needed. Promising targets are histone deacetylases (HDACs), since they regulate posttranslational protein acetylation. Over a thousand proteins are reversibly acetylated, and acetylation critically influences aberrant intracellular signaling pathways in asthma and COPD. The diverse set of selective and non-selective HDAC inhibitors used in pre-clinical models of airway inflammation show promising results, but several challenges still need to be overcome. One such challenge is the design of HDAC inhibitors with unique selectivity profiles, such as selectivity towards specific HDAC complexes. Novel strategies to disrupt HDAC complexes should be developed to validate HDACs further as targets for new anti-inflammatory pulmonary treatments.

## 1. Introduction

Inflammatory pulmonary diseases are among the most common health problems worldwide. Currently, approximately 339 million people suffer from asthma, which results in 250 thousand preventable deaths annually [1,2]. Similar numbers have been reported for Chronic Obstructive Pulmonary Disease (COPD). About 384 million people were estimated to have COPD and 3 million people died because of it in 2010 [3,4,5,6]. COPD is a slow and inevitably progressing disease that, most notably in the later stages, is very debilitating [7]. Inflammatory pulmonary diseases, such as asthma and COPD, are generally chronic and can severely reduce the quality of life. However, to date, there is only a limited number of effective therapeutics available for these diseases. From this perspective, novel molecular mechanisms that can be targeted by novel therapeutics need to be identified in order to allow for the development of more sophisticated treatment strategies [1,2,3,4,8].

Inflammation in asthma and COPD is mediated by multiple regulatory mechanisms. On the molecular level, cellular signaling in inflammation is critically regulated by post-translational modifications (PTMs). The dynamic process of addition or removal of PTMs by respective enzymes influences the properties of the involved protein. The most well-studied PTM is acetylation, in which an acetyl-group is added to a lysine residue by a histone acetyl transferase (HAT) or removed by a histone deacetylase (HDAC). As an important example, acetylation of histones facilitates transcriptional activation either by neutralizing the ionic interaction between DNA and the histones or by forming a binding site for chromatin remodeling proteins and transcription factors [9,10]. Disruption of this process results in abnormal gene expression that contributes to the pathogenesis of asthma and COPD. Restoration of protein acetylation thus offers an interesting avenue in the search for new treatments for asthma and COPD.

In this review, we aim to describe the molecular mechanisms in asthma and COPD, wherein protein acetylation plays a key role. Based on these mechanisms, we aim to define the potential utility of histone deacetylase inhibitors (HDACi) as therapeutics in inflammatory pulmonary diseases and summarize the recent development of isoform selective inhibitors. Lastly, we intend to complement recent reviews covering developments in targeting HDAC complexes with small molecule inhibitors and analyze which complexes are important for inflammation. An important future challenge will be to develop tools that selectively interfere with HDAC complexes involved in inflammation.

## 2. Pathogenesis of Asthma

### 2.1. Cellular Mechanisms of Asthma

**Figure 1:**
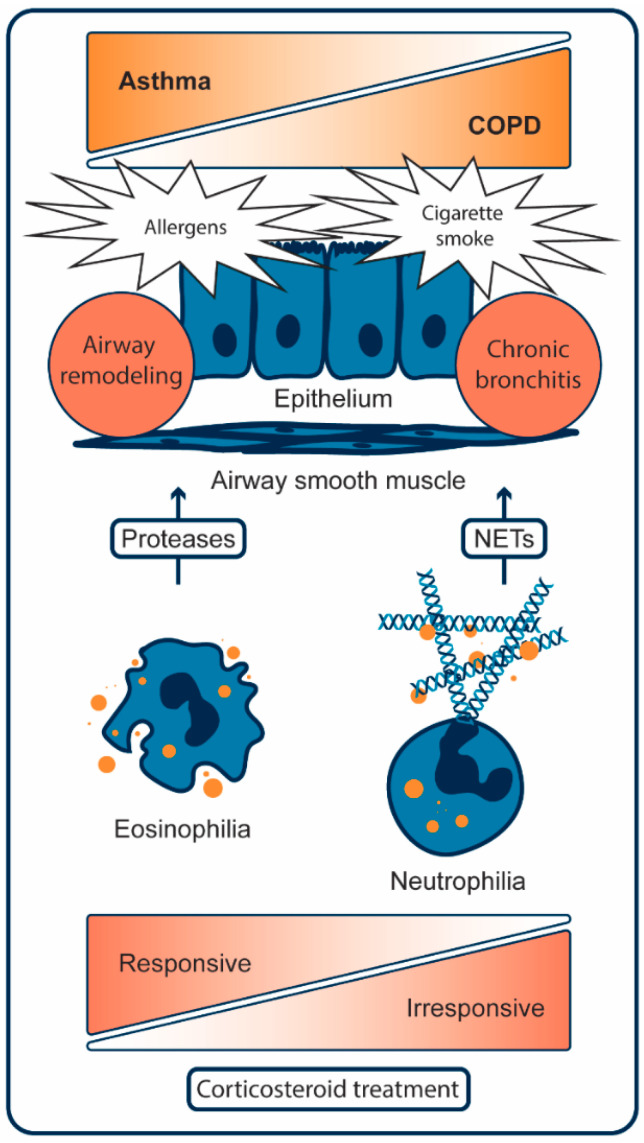
Key cellular mechanisms in asthma and COPD. In asthma, allergen exposure results in airway remodeling through a persistent type-2 inflammatory response. This response increases eosinophils and damages lung tissue by released proteases from granules (orange dots). Eosinophilic asthma can be suppressed with corticosteroid therapy. In COPD, cigarette smoke results in chronic bronchitis and emphysema through a persistent non-type 2 inflammatory response. This response increases neutrophils and damages lung tissue by released proteases from granules (orange dots) and DNA in neutrophil extracellular traps (NETs). Corticosteroid treatment is largely ineffective in suppressing neutrophilic inflammation [23,24].

Asthma is a multifactorial disease with a clear inflammatory component [11]. The pathophysiological mechanisms of inflammation in asthma can broadly be divided into T-helper 2-high (type 2) and T-helper 2-low (non-type 2) subtypes [12]. Hallmarks of T-helper 2-driven inflammation are the presence of the cytokines IL-4, IL-5 and IL-13 and the involvement of mast cells, eosinophils and IgE-producing B cells [13]. These cells influence each other with the mentioned cytokines to mount an inflammatory response. For example, IgE-dependent activation of mast cells triggers them to release their granules containing histamine and tryptase and to generate pro-inflammatory cytokines such as prostaglandin D_2_, cysteinyl leukotrienes (LTC_4_ and LTD_4_) and adenosine [14,15]. Mast cells are also key sources of the allergy associated cytokines IL-4 and IL-5 [16]. IL-4 aggravates CD4^+^ T cells to differentiate into T-helper type 2 cells and further enhances IgE-mediated immune responses and inflammatory cell recruitment [17,18] and IL-5 induces generation of novel eosinophils in the bone marrow and their subsequent chemotaxis towards inflamed lung tissue leading to airway eosinophilia [19]. Eosinophils are the key effector cells that cause most of the tissue damage through release of proteases; Figure 1. Distinctively, where type 2 inflammation can be suppressed by inhalation of corticosteroids, non-type 2 inflammation is largely unresponsive to corticosteroid therapy [20].

The molecular and cellular mechanisms that underpin non-type 2 inflammation in asthma are less clear, but may principally involve neutrophils and high levels of IL-17 instead of eosinophils and IL-4, IL-5 and IL-13 [20,21]. Airway neutrophilia in non-type 2 asthma is the result of an extended lifespan or failed clearance of apoptotic neutrophils and has mostly been observed in severe cases of asthma [22]. As a plausible pathological mechanism, neutrophils form neutrophil extracellular traps (NETs) that may damage nearby lung tissue and impede the resolution of inflammation; Figure 1 [23,24]. NET formation starts with disassembly of the nuclear envelope, followed by DNA decondensation and rupture of the plasma membrane to expel web-like structures of DNA decorated with histones, proteases and peroxidases [25]. Such NET formation, or NETosis, kills the neutrophil. Additionally, neutrophils are known to degranulate and eject DNA to create NETs extracellularly and leave an anucleated cell body, called a cytoplast, behind [26]. Cytoplasts activate lung dendritic cells to promote differentiation of CD4^+^ T cells into T-helper 17 cells, which express IL-17 [26]. IL-17 stimulates epithelial cells to produce IL-8 [27] and thus leads to an increase in the production and recruitment of neutrophils, thereby creating a vicious cycle of neutrophil induced lung damage.

Regardless of the type of inflammation, asthma is further pathologically characterized by abnormal structural changes in the airway epithelium and submucosa that lead to airway obstruction, airway hyperresponsiveness and airway mucosal inflammation [28,29]. The most apparent change in the epithelium is goblet cell hyperplasia [30]. Changes in the submucosa include smooth muscle cell hyperplasia and subepithelial fibrosis [28]. Whether airway remodeling is driven or just modified by inflammation is unclear, but it is clear that inflammation increases the susceptibility to exacerbation of the disease. Therefore, attenuating the persistent expression of inflammatory molecules required for the recruitment and activation of neutrophils, eosinophils and T lymphocytes offers a way to control asthma.

### 2.2. Role of HDACs in Asthma

The acetylation status of proteins in asthma is reported to be maintained by an intricate balance of HATs and HDACs. There are four main classes of HDACs. The class III HDACs use nicotinamide adenine dinucleotide (NAD^+^) as cofactor. These HDACs are named sirtuins and can reside in the nucleus, mitochondria or cytoplasm, depending on the isoform [31]. Class I, II and IV HDACs depend on a zinc ion in their active site for catalytic activity [32,33]. Class I HDACs, HDAC1, 2, 3 and 8 are generally localized in the nucleus. Class IIA HDACs include HDAC4, 5, 7, 9 and class IIB harbors HDAC6 and 10 and have mostly cytoplasmic functions [34,35]. Class IV comprises the elusive HDAC11, which is only found in immune cells [36]. The family of proteins that has HAT activity is even more diverse.

HAT activity and the acetylation state of histones is increased in biopsies from children and adults suffering from asthma [37,38]. Acetylation of histone 3 lysine 27 (H3K27ac) and mono-methylation of H3K4 are essential in maturation of progenitor CD4^+^ cells into T-helper 2 cells and thus for the pathophysiology of asthma [39]. Additionally, a study found a small decrease in the expression of HDAC1 and HDAC2 in bronchial biopsies of asthma patients compared with healthy individuals [38]. Alveolar macrophages from asthmatic patients are also found to harbor a decreased deacetylase activity related to a decreased expression of HDAC1. The same decrease was not found in circulating blood monocytes thus confirming the notion that aberrant HDAC expression is localized at the site of inflammation [40]. The decreased expression of HDAC1 is also proposed to be a biomarker for severe asthma [41]. The extent of HDAC1 reduction is speculative, since a slight increase in HDAC1 in patients suffering from severe asthma compared with regular patients has also been observed [42]. The expression levels of other HDACs has so far not been adequately characterized. In summary, the expression of HDAC1 and HDAC2 in asthma is either slightly increased or decreased, and HAT activity is slightly increased.

In addition to differential HDAC expression, single nucleotide polymorphisms in both HDAC1 and HDAC2 have been observed in patients with asthma [43]. A polymorphism in HDAC1 was shown to have a significant relationship with asthma severity and its presence was associated with lung function improvements in response to inhaled corticosteroid treatment in childhood asthmatics [43]. However, the functional role of the polymorphism was not elucidated.

## 3. Pathogenesis of COPD

### 3.1. Cellular Mechanisms of COPD

Increased oxidative stress as a result of the inhalation of noxious particles, particularly those derived from cigarette smoke, is a key driving mechanism in the pathogenesis of COPD and leads to the destruction of lung parenchyma [44]. This process is mediated by cytotoxic CD8^+^ T cells, macrophages and neutrophils. These cells release various proteases, such as elastase and matrix metalloproteinase 9 and 12, that break down connective tissue in the lung parenchyma to result in emphysema [45]. The proteases, together with epidermal growth factors, also induce goblet cell hyperplasia and enhance mucus production and secretion to result in chronic bronchitis [46]. The release of proteases by neutrophils in COPD is, similar to non-type 2 inflammation in asthma, in part the result of NET formation, see Figure 1, mediated by IL-8 [23,24]. In addition to elucidation of the cellular mechanisms, research in COPD has focused at unravelling the intra-cellular pathways that are activated in the inflammatory cells in COPD. In particular, the mechanism behind corticosteroid insensitivity and strategies to revert it have gained attention. These strategies converge at restoring HDAC2 activity, as will be discussed in the next paragraph.

### 3.2. Role of HDACs in COPD

Patients suffering from severe COPD are found to express less than 5% of the HDAC2 that nonsmokers do [47]. This decreased expression of HDAC2, which deacetylates histone 4 (H4) at the IL-8 promotor, correlates with disease progression and is proposed as biomarker for disease severity [48,49]. Additionally, HDAC5 and 8 were expressed to a lesser extent, SIRT1 activity is decreased, HDAC1, 3, 4, 6 and 7 expression levels were unchanged and the expression and activity of HDAC9, 10, 11 and the other sirtuins is unknown [49,50]. Altogether, these studies indicate that HDAC expression and activity is altered in COPD.

Whether the observed decrease in HDAC2 expression in COPD is truly part of the etiology of the disease is difficult to assess. A study that exposed rats to cigarette smoke found a decrease in the expression of HDAC2 after 3 days but not after 8 weeks of cigarette exposure [51]. The initial decrease after 3 days can be the result of a cascade of reactions originating from oxidative stress. Oxidative stress is mediated by a combination of superoxide anions and nitric oxide, derived from cigarettes smoke, to provide peroxynitre [52,53]. The formed peroxynitre is anticipated to nitrate tyrosine residues on HDAC2 [53]. These nitrated residues then trigger ubiquitination and subsequent proteasomal degradation of HDAC2 [54]. Furthermore, reactive oxygen species activate phosphoinositide-3-kinases, the class I PI3K-δ isoform in particular. The downstream kinase AKT, or PI3K-δ itself, phosphorylates the serine residues on HDAC2, also leading to ubiquitination and proteasomal degradation [55]. This is an explanation for the initial decrease in HDAC2 after 3 days. In the weeks after the initial decrease in HDAC2, synthesis of HDAC2 is upregulated to counteract the proteasomal degradation and to return HDAC2 levels to those found pre-smoke exposure. This would mean that the observed long-term reduction in HDAC2 expression in COPD patients has a different origin.

The group led by Barnes identified HDAC2 as an important regulator of the glucocorticoid receptor (GR) pathway. The GR receptor moves to the nucleus upon activation by a glucocorticoid. There the GR is deacetylated by HDAC2, which allows the GR to form a protein–protein complex that represses the NF-κB pathway [56,57,58]. This attenuates inflammation in asthma patients taking corticosteroid medication [59]. Conversely, a decrease in the expression of HDAC2, through activation of PI3K-δ during oxidative stress, abolishes the effect of glucocorticoids in patients with COPD [60]. The GR is then not deacetylated and thus cannot repress NF-κB. As further proof, restoration of HDAC2 activity by inhibition of PI3K-δ by either nortryptiline or a low-dose of theophylline helps to alleviate glucocorticoid irresponsiveness in COPD [61,62,63].

Interestingly, glucocorticoid irresponsiveness might also result from the upregulation of the β isoform of the GR. Upon treatment with glucocorticoids, transcription of the GRβ isoform is upregulated, especially in the case of concomitant exposure to IL-17, which is increased in COPD [64,65,66,67]. GRβ directly interferes with the promotor activity of HDAC2 to result in a decreased expression of HDAC2 [68]. In this way, the observed reduced level of HDAC2 expression is mainly the result of glucocorticoid treatment, in combination with an increased level of IL17, and not a direct effect of inhalation of noxious particles. This would constitute an acquired form of resistance towards glucocorticoids. The previously mentioned HDAC expression data in COPD patients taking glucocorticoids was not corrected for the potentially confounding variable of glucocorticoid treatment.

Altogether, the group led by Barnes identified HDAC2 as an important player in activating the anti-inflammatory response upon activation of the GR pathway with glucocorticoids. Additionally, inhalation of noxious particles is linked to an acute decrease in the levels of HDAC2 and these lower levels of HDAC2 might be maintained by continuous smoke exposure and glucocorticoid treatment.

## 4. The Role of NF-κB Acetylation in Asthma and COPD

The NF-κB signaling pathway plays a central but complex role in asthma and COPD and in inflammation in general. The pathway converges on NF-κB and related transcription factors, which bind to the promotors of pro-inflammatory genes and increase their expression. Importantly, the activity of the NF-κB protein complex is influenced by acetylation. NF-κB contains seven lysine residues that can all undergo the process of acetylation and deacetylation in a site-specific manner. The CBP/p300 acetyltransferases play a major role in acetylating the seven lysine residues on NF-κB [69]. Although HDAC1 and HDAC2 are known to interact with NF-κB, deacetylation of the lysine residues is mostly under the control of HDAC3 [70]. To be more precise, HDAC3 deacetylates lysine 122, 123, 314 and 315, which in their acetylated form negatively regulate NF-κB activity [71]. This is further supported by the finding that HDAC3-deficient macrophages induce only half of the genes linked to LPS-induced inflammatory gene expression [72].

Acetylation of lysines 122 and 123 inhibits binding towards DNA [70]. On the other hand, acetylation of lysines 218 and 221 on subunit p65 (RelA) prevents interaction with the inhibitory protein IκBα, allowing translocation towards the nucleus [73]. Additionally, lysine 310 acetylation is needed for full transcriptional activity of p65, possibly by being a binding site for bromodomain containing proteins that direct transcription [73,74]. Intriguingly, acetylation of lysines 314 and 315 direct NF-κB towards specific promoter regions [75]. As an example of how acetylation controls the binding site of NF-κB in asthma, the bromodomain protein BRD4 has a binding site for the acetylated NF-κB subunit p65 as well as a binding site for acetylated histones H3k9 and H3k27. In this way, BRD4 directs the transcription factor to specific locations along the chromatin to increase the transcription of genes related to proliferation and inflammation [76]. This means that the transcriptional activity of NF-κB depends on acetylation. More importantly, the affinity towards different promoters and interaction with transcriptional proteins changes upon acetylation of NF-κB.

## 5. HDACi in Asthma and COPD

Currently over 100 HDACi are in clinical trials for cancer therapy [77] and various reports suggest that HDACi could also be effectively used to modulate inflammatory diseases, in part because the underlying disease mechanics in cancer overlap with inflammation [78,79]. Moreover, the anti-inflammatory effects of HDACi are seen at concentrations 10–100-fold lower than their cell-killing properties observed in cancer [80]. Hence, using HDACi for the treatment of inflammatory diseases, like asthma and COPD, is a topic of current research.

Given that HDAC3 is a positive regulator of NF-κB mediated inflammation, inhibitors of HDAC3 have been proposed as novel therapeutics to combat inflammation in COPD and asthma [5]. In support of this, selective inhibition of HDAC3 with the inhibitor RGFP966 in LPS/IFN-γ stimulated macrophages attenuated the NF-kB transcriptional activity and demonstrated anti-inflammatory effects [81]. However, the acetylation status of NF-κB p65, histone H3 and histone H4 was unaffected, and the most promising effects were seen at a relatively high concentration of 10 µM, at which RGFP966 also inhibits HDAC1 and HDAC2. On the other hand, siRNA-mediated downregulation of HDAC3 reduced the expression of the pro-inflammatory genes IL-1β, IL-6 and IL-12b up to 60%. This may point to an important structural role for HDAC3 in inflammation instead of its catalytic role in deacetylation. Whether selective pharmacological inhibition of HDAC3 in in vivo models of asthma or COPD will be beneficial remains to be seen. Knocking-out HDAC3 might be a more promising approach.

**Figure 2:**
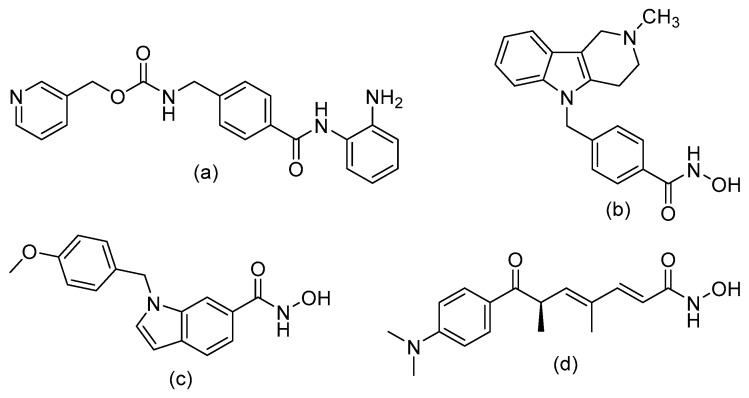
Histone deacetylase inhibitors used in in vivo models of asthma or chronic obstructive pulmonary disease. (**a**) Structure of the HDAC1/2/3 selective inhibitor entinostat. (**b**) Structure of the HDAC6 selective inhibitor tubastatin A. (**c**) Structure of the HDAC8 selective inhibitor PCI-34051. (**d**) Structure of the non-selective HDAC inhibitor trichostatin A.

Nonetheless, inhibition of HDAC1, 2 and 3 by entinostat (Figure 2A) in LPS/IFN-γ induced macrophages in a COPD mouse model led to increased acetylation of NF-κB, increased translocation towards the anti-inflammatory IL-10 promoter and subsequent increased expression of IL-10 [82]. Inhibition of HDAC1, 2 and 3 furthermore reduced inflammation through decreased pro-inflammatory cytokine expression of IL-8, IL-6 and IL-1β (Table 1). In conclusion, entinostat clearly reduced cigarette smoke-induced airway inflammation in mice and therefore shows potential for the treatment of COPD.

In addition to HDAC1/2/3, HDAC6 and HDAC8 are important in the regulation of cellular processes in inflammation. For example, HDAC8 is known to deacetylate cortactin. This promotes actin filament polymerization and subsequent smooth muscle contraction, which plays an important role in airway inflammation and remodeling [83]. Selective inhibition of HDAC8 with the inhibitor PCI-34051 (Figure 2B) has been shown to attenuate airway hyperresponsiveness and inflammation and counteract airway remodeling to a certain extent [84]. Another HDAC that is important in airway remodeling is HDAC6, which primary function is the deacetylation of α-tubulin. Tubulin is a major component of the cytoskeleton and is thereby involved in cell motility [85,86]. Acetylation of α-tubulin leads to stabilized microtubules, and thereby decreases cellular motility. The importance of HDAC6 is exemplified by knock-out mouse models that display impeded macrophage migration and motility [87]. Fibroblasts also have reduced motility upon inhibition of HDAC6 [87,88]. Inhibiting HDAC6 could in this way attenuate airway remodeling in inflammation. Indeed, upon treatment of asthmatic mice with the HDAC6 selective inhibitor tubastatin A (Figure 2C), airway hyperresponsiveness and inflammation were mitigated along with a decrease in airway remodeling markers [84]. Additionally, mice that were exposed to cigarette smoke and injected with tubastatin A were protected from cigarette smoke-induced mucociliary clearance disruption [89]. To summarize, HDAC6 and HDAC8 could be targeted with selective HDACi to combat airway remodeling and inflammation in both asthma and COPD.

More general inhibition of multiple HDACs in in vivo models of airway inflammation also point towards a beneficial anti-inflammatory effect. The non-selective HDACi TSA (Figure 2D) reduces inflammation in human precision cut lung slices and in in vivo mouse models [90,91]. TSA treatment reduced inflammation by reducing the expression of IL-17 and T-helper 17 cell numbers, while increasing Treg cell activation. Furthermore, the expression of TGF-β in the bronchoalveolar lavage fluid increased following HDAC inhibition in a mouse model of asthma [84]. These changes were associated with increased acetylation at the TGF-β promoter. 

The anti-inflammatory effects of TSA have also been postulated to be partly the result of enhanced apoptosis in neutrophils and eosinophils through activation of the c-jun-*N*-terminal kinase pathway involving caspases 3 and 6 [92]. TSA was shown to have an additive effect on apoptosis in eosinophils following glucocorticoid treatment and TSA antagonized glucocorticoid-induced neutrophil survival [92]. Similar effects have been observed for other non-selective HDACi, which dose-dependently switch neutrophil death from NETosis to apoptosis [93]. This could have important implications for the timely execution of neutrophil apoptosis which has been reported to be dysregulated in severe asthma [22]. Yet, non-selective HDACi also induce apoptosis in macrophages by decreasing the expression of the anti-apoptotic Bcl-2–like protein Bfl-1 in macrophages [94]. Macrophages play an important role in the removal of apoptotic cells [95]. Increased apoptosis of eosinophils and neutrophils without their timely clearance by macrophages will result in disintegration of the apoptotic cells, causing damage to lung tissue and propagation of the inflammatory response [96]. Yet, in addition to macrophages, airway epithelial cells are also capable of phagocytosing apoptotic eosinophils [97] and these epithelial cells, like many other cell-types, do not undergo apoptosis following HDACi treatment [98].

In conclusion, both isoform selective inhibition, as well as more general inhibition of HDACs, seem to offer treatment options in asthma and COPD (Table 1). Current investigations aim at defining the importance of specific HDAC isoforms in terms of their structural role or their catalytic role. This provides potential novel starting points for drug discovery. Inhibition of the catalytic activity of HDAC6 and HDAC8 could potentially alleviate airway remodeling and decrease inflammatory cell motility. In contrast, complete knock-out of HDAC3, as described in the beginning of this paragraph, might be required to attenuate airway inflammation.

## 6. Design of Selective HDACi Targeting the Catalytic Site

The clear beneficial effects of selective HDACi in the described models of asthma and COPD advocate the further development of selective inhibitors. An overview of ongoing medicinal chemistry efforts in this area is provided in this section. The general pharmacophore of HDACi comprises a cap group that interacts with the rim of the enzyme’s cavity that is connected via a hydrophobic spacer to a zinc binding group (ZBG). The largest group of HDACi, including three of the four FDA-aproved drugs, contain a hydroxamic acid as a bidentate zinc chelator. The hydroxamic acid moiety can target HDACs from various classes (like TSA, Figure 2D), but has also been used to engineer the HDAC6 selective inhibitor Tubastatin A (Figure 2B) and the HDAC8 selective inhibitor PCI-34051 (Figure 2C). The selectivity difference between these hydroxamic acid HDACi is poorly understood but is under active investigation [99]. Additionally, compounds with an *o*-aminoanilide ZBG (like entinostat, Figure 2A) have been developed, and these mainly target HDAC1/2 and 3. The reason for this specificity between the ZBGs is that HDAC1/2 and 3 have an additional 14 Å-wide cavity in the active site, called the foot pocket [100]. HDAC8 and the other HDACs lack a foot pocket, thus conferring insensitivity towards these bigger ZBGs. The foot pocket is thought to accommodate the acetate byproduct that is generated during the hydrolysis of the acetyl group. Furthermore, the foot pocket is hypothesized to connect to an acetate release tunnel, whose exit is controlled by gate-keeping aromatic amino acids that only transiently open to allow acetate to escape [101]. It is, therefore, attractive to consider HDACs as dynamic scissors, efficiently cutting acetyl groups off lysine residues. More importantly, the acetate cavity has enabled the design of selective inhibitors targeting either HDAC1 and HDAC2 or HDAC3 through the modification of the *o*-aminoanilide ZBG.

### Structure–Activity Relationship of Reported o-Aminoanilides for Selective Inhibition of HDAC1/2/3

**Figure 3:**
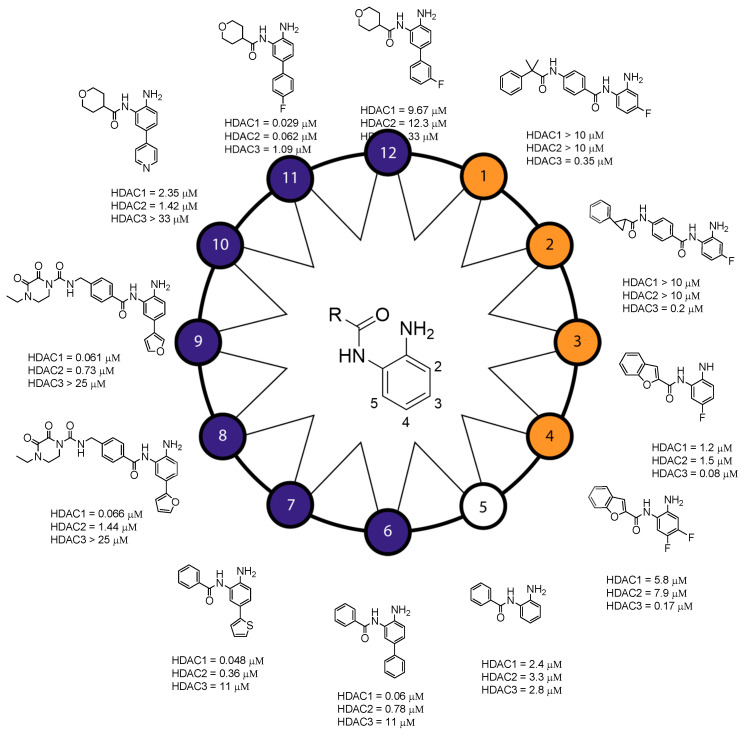
Structure–activity relationship of reported *o*-aminoanilides. In brief, fluor atoms in position 3 or 4 give rise to HDAC3 selective inhibitors (compounds **1**–**4, orange circles**) and aromatic groups in position 4 (compounds **6**–**12, purple circles**) push selectivity towards HDAC1 and 2. The unsubstituted *o*-aminoanilide (compound **5, white circle**) inhibits HDAC1,2 and 3 with equal potency. Compounds with substitutions at either **2** or **5** (not shown) are unable to inhibit HDACs at relevant concentrations.

Crystal structures of an *o*-aminoanilide in complex with an HDAC show that the amino and anilide groups together chelate the zinc ion through an unusual seven-membered ring (see Figure 3, middle). Introducing a group on the amine, or modifying the anilide accordingly leads to a loss of potency [102,103]. However, exchanging the amino group for a hydroxyl does not make a difference [102,104], indicating that both are able to interact with the zinc ion. Figure 3 shows the effects of substitutions on the anilide ring on the HDAC isoenzyme inhibitory selectivity. Substitutions at position 2, ortho to the amine, or position 3, meta to the amine, give rise to interesting selectivity profiles. Compounds bearing a fluorine atom in position 3 show an outstanding selectivity for HDAC3 inhibition, like compound **1** and **2** [105]. Replacing fluorine with a chlorine leads to a decrease in potency similar to replacement with the electron-donating methyl and methoxy groups [102,106]. Collectively, strong but small electron-withdrawing groups in position 3 will lead to potent and selective inhibition of HDAC3, while either larger or electron-donating groups will decrease it. Contrarily, compounds with a fluorine atom or methyl group in position 2 completely lose their ability to inhibit HDACs [102,107]. The same is true for modifications in position 5, meta to the amine, but on the side of the anilide; a fluorine in that position leads to a significant loss in potency [107]. Substituents in position 2 or 5 probably sterically hinder the amino and anilide groups, thereby preventing proper coordination towards the zinc ion.

Concerning position 4, para to the amine, compound **3** with a fluorine atom in that position is also reported to selectively inhibit HDAC3, with a respective 15- and 19-fold higher concentration needed for the inhibition of HDAC1 and 2 [107]. An even better selectivity profile is obtained by combining a fluorine in position 2 with one in position 4, as illustrated in compound **4** [107]. In comparison, the unsubstituted analogue (compound **5**) has IC_50_ values of 2.4 μM, 3.3 μM, and 2.8 μM for HDAC1/2/3, respectively [104]. Interestingly, aromatic substituents in position 4 lead to selective inhibition of HDAC1 and HDAC2 over HDAC3 [104,108,109]. For example, compound **6**, in which a phenyl ring is introduced in position 4, has corresponding IC_50_ values of 0.06 μM, 0.78 μM and 11 μM. Other investigations confirmed that aromatic groups in the 4 position, such as 2-thienyl (compound **7**) [104], 2-furyl (compound **8**) [108], 3-furyl (compound **9**) [108] and 4-pyrimidinyl (compound **10**) [103], result in preferential binding to HDAC1 and 2. Insertion of an oxygen-, carbonyl-, or ethyl-linker between the aromatic substituents and the *o*-aminoanilide ring leads to a decrease in potency (not shown in Figure 3) [104]. Some experiments were conducted with *o*-aminoanilide derivatives with a carboxylic acid or a carboxamido group in the 4 position, but this reduced their ability to inhibit HDAC1 [104]. Potential inhibition of other HDACs was in this case unfortunately not investigated. Other changes in position 4 are based on the further modification of the aromatic rings in position 4. Most of these substituents (e.g., 4-chlorophenyl, 4-trifluoromethylphenyl) did not improve selectivity. Compound **11**, however, having a 4-fluorophenyl in position 4, is promisingly potent, with an IC_50_ of 0.029 μM for HDAC1. A 2-fold higher concentration is needed for inhibition of HDAC2, and a 38-fold higher concentration for HDAC3 inhibition [103]. Interestingly, 3-fluorophenyl substituents (compound **12**) decrease potency, with a 333-fold increase in the IC_50_ for HDAC1, 198-fold for HDAC2, and even no inhibition of HDAC3 at the measured concentrations [103]. From the findings above it becomes clear that small differences in the substitution pattern of *o*-aminoanilides result in substantial differences in potency and selectivity. In conclusion, a fluorine in position 3 confers selectivity towards HDAC3, while aromatic substitutions in position 4 only fit in the foot pocket of HDAC1 and HDAC2 and thereby confer selectivity towards HDAC1 and HDAC2. Selectivity between HDAC1 and HDAC2 has so far not been described for *o*-aminoanilide HDACi.

## 7. Targeting HDAC Complexes

### 7.1. Class I HDAC Complexes

The next step in the design of selective HDACi would be to go from structure-activity relationships on single enzymes to structure-activity relationships on HDACs as part of a broad set of multi-protein complexes with diverse functions. HDAC1 and 2 form the catalytic core of the complexes Sin3 (switch-independent 3), NuRD (nucleosome remodeling and deacetylase), CoREST (co-repressor of REST), MIER (mesoderm induction early response), RERE (arginine-glutamic acid dipeptide repeats), and MiDAC (mitotic deacetylase). The structures of HDAC1 and HDAC2 are very similar, and as such, are recruited interchangeably towards the same complexes [110]. Additionally, HDAC1 and HDAC2 can form hetero- and homodimers and dimerization enhances their activity [111]. HDAC3 gets recruited exclusively towards the SMRT/NCoR (nuclear receptor co-repressor) complex. Furthermore, HDAC8 does not form a complex with other proteins [112]. Complex formation of HDAC1/2/3 is needed for maximum deacetylase activity and provides directionality towards specific places of the genome. Additionally, HDAC complex formation influences the binding preference of HDACi. In the case of *o*-aminoanilide HDACi, one study showed that these inhibitors preferentially bind to the HDAC3/NCoR complex rather than the NuRD, CoREST or MiDAC HDAC complexes [113]. The Sin3/HDAC1/2 complex was shown not be targeted by these HDACi at all, which is largely due to thermodynamic rather than kinetic reasons [114]. This interesting finding prompts us to review HDAC protein complexes important in inflammation, because this is relevant for the development of inhibitors. Also, novel examples will be given regarding drugs developed to specifically target these complexes since the most recent review [115].

### 7.2. The Sin3 Complex

#### 7.2.1. Structure of Sin3

Sin3 acts as a scaffold protein for HDAC1 and HDAC2 and other proteins that guide HDACs to their target [116]. Humans harbor two isoforms of Sin3—Sin3A and Sin3B—that are expressed ubiquitously throughout the body. The scaffolds are considered to be master gene regulators that are highly conserved throughout the phylogenetic tree of life. They show about 57% structural homology and are found to bind both the same and divergent transcription altering proteins [116,117]. Elucidating the complete structural properties and targets of the Sin3 protein complex is currently a topic of active research [118].

#### 7.2.2. Roles of Sin3

Classically, Sin3 is the prototypical DNA repressor complex since it directs HDACs towards the chromatin, specifically towards histones H3 and H4. Their subsequent deacetylation increases the interaction of the chromatin with the histones and yields Sin3 mediated gene repression. However, in Sin3 knockout studies in fruit flies, yeasts and mice, both transcriptional up- and down-regulation have been observed [119,120,121,122]. As a consequence, the Sin3 complex has been defined as a co-repressor, co-activator and general transcription factor in recent literature [123]. How Sin3 increases gene transcription is currently unknown. It is anticipated to be important in regulation of crucial cellular functions. As an example, a study performed in fruit flies showed that Sin3 deficient cells have a delayed G2 phase. These cells also exhibit increased expression of genes related to energy metabolism and displayed increased mitochondrial mass [121]. Additionally, Sin3 is important in repressing cell division [124]. This is exemplified by studies linking Sin3 deficiency to an increase in cell invasion and tumorigenesis [125]. More examples of the effects on cellular proliferation, apoptosis, differentiation and cell cycle regulation are reviewed elsewhere [118,122]. Taken together, these studies suggest that the Sin3 complex is important in the regulation of genes important in cell maturation.

#### 7.2.3. Examples of Sin3 Targeting

**Figure 4:**
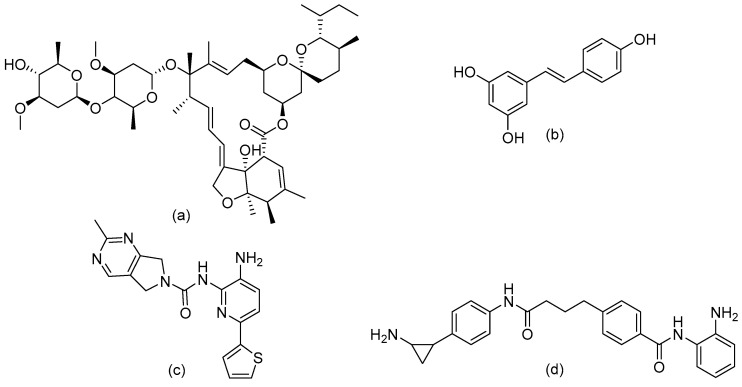
Structures of histone deacetylase complex inhibitors. (**a**) Structure of ivermectin, a macrocyclic lactone derived from *Streptomyces avermitillis* used to treat parasitic infection in human and veterinary medicine. Ivermectin is also shown to selectively inhibit Sin3 complex formation. (**b**) Structure of resveratrol, a dietary supplement shown to decrease MTA1 expression. (**c**) Structure of Rodin-A, an example of an HDACi with relative selectivity for inhibition of CoREST. (**d**) Structure of corin, a bivalent HDAC1/2/3 and LSD1 inhibitor directed against the CoREST complex.

Targeting the Sin3 complex or subsets of the Sin3 complex in cancer might be beneficial because of its importance in cellular development [126]. Indeed, inhibition of Sin3 complex formation in breast cancer models shows potential [127]. The researchers used the antiparasitic drug ivermectin (Figure 4A), which blocks the formation of Sin3 by occupying a protein–protein binding site [127]. Mutations were made in proteins of the Sin3 complex to identify important residues for protein–protein interaction. They showed that preventing protein–protein binding with ivermectin is indeed a way to impair complex formation. Additionally, the efficacy of ivermectin for the treatment of asthma has been tested in a mouse model. Ivermectin significantly diminished the production of the cytokines IL-4, IL-5 and IL-13 and reduced the recruitment of neutrophils and eosinophils [128]. However, ivermectin has many different modes of action, including through interaction with ligand-gated channels [129], and it is therefore unclear to what extent disruption of the Sin3 complex contributes to the observed anti-inflammatory effects.

### 7.3. The NuRD Complex.

#### 7.3.1. Structure of the NuRD Complex

The core of the NuRD complex consists of a dimer of the histone binding proteins MTA1/2/3, four copies of RBBP4/7 and two copies of either HDAC1 or HDAC2 or a mix of HDAC1 and HDAC2. Additionally, two copies of the methyl binding domain proteins MBD2/3 can join in to mediate the association with MTA1/2/3 and modify deacetylase activity [130]. The nuclear zinc-finger transcriptional repressor p66-α (GATAD2A) and p66-β (GATAD2B) also directly interact with MBD proteins [131]. Finally, Mi-2α/β, also known as CHD3/4, which is a chromodomain-helicase-DNA-binding protein that uses ATP to modify the chromatin structure is also able to bind to the complex [132]. Recruitment of the two copies of HDAC1/2 towards the complex is mediated by the dimeric ELM2-SANT domain of MTA1/2/3, accompanied by the four equivalents of RBBP4/7 towards the C terminus of MTA1/2/3 [115,133,134,135].

#### 7.3.2. Roles of NuRD

Overall, NuRD is regarded as an important mediator during the developmental stages of life, playing important roles in cell cycle progression, DNA repair and chromatin remodeling [136]. Hence, targeting this complex could be beneficial in regenerative medicine or cancer. For example, it has been shown that Mi2-β has intrinsic activities on its own, as it associates with a CD4 gene transcription enhancer and the HAT p300 to increase CD4 gene expression needed in T-cell development [137]. Additionally, different MTA1/2/3 subtypes are found in distinct complexes where they have different functions [138]. For example, MTA1, which is upregulated in a variety of tumors [139], has also been shown to be a regulator of inflammatory homeostasis [140]. Researchers found that MTA1 transcription was upregulated in LPS induced cells through an NF-κB controlled mechanism. They also found fewer MTA1:HDAC2 repressor complexes near LPS inducible genes such as IL-1β, TNF-α and MIP2. Furthermore, expression levels of IL-1β and TNF-α in macrophages from LPS induced MTA1 knockout mice were 6.7- and 3.72-fold higher than in wild-type. This is indicative of the double role of MTA1, repression of inflammatory genes under basal conditions but an inflammatory mediator upon NF-κB activation [140]. Taken together, these studies suggest an enhancing role for MTA1 in inflammation and a repressing role for MTA1:HDAC2 of inflammatory genes via deacetylation or occupation of the promoters. Therefore, MTA1 is a potential drug target to suppress inflammation in inflammatory disease [141]. More research regarding this interaction is needed for a precise determination of the mechanisms.

#### 7.3.3. Targeting NuRD

Resveratrol (Figure 4B), a dietary supplement found in grapes, decreases expression of MTA1 in prostate cancer cells [142]. This in turn decreases the amount of MTA1:HDAC1 complexes. How resveratrol decreases expression is unknown. Additionally, MTA1 inhibition by pterostilbene, a compound that has a similar structure and function to resveratrol, in combination with a HDACi has shown to be effective in a prostate cancer mouse model [143]. Effectivity in models of inflammation remains to be investigated. Selective targeting of the many different NuRD complexes is difficult, since there are several possible combinations between the subunits and structural information of each subunit is incomplete.

### 7.4. The CoREST Complex

#### 7.4.1. Structure and Roles of the CoREST Complex

The structural information on the CoREST complex is the most complete, probably because CoREST is one of the smallest complexes known to form complexes with HDACs. The complex consists of one HDAC1 and HDAC2, one CoREST1/2/3 scaffold protein and one lysine-specific histone demethylase 1(LSD1) [144,145]. The CoREST scaffold subtypes each have different effects on the other proteins in the complex [146]. It is anticipated that the CoREST protein binds to HDACs using a ELM2-SANT domain and does not form dimers [147]. The general consensus is that the CoREST complexes are important in regulation of neuronal genes [148]. Furthermore, CoREST is shown to be important in inducing and maintaining specific neuronal subtypes [149]. This could conceivably be used to develop compounds to combat neurodegenerative diseases such as Alzheimer’s and Parkinson’s diseases.

#### 7.4.2. Targeting CoREST

Knowing that HDACi have varying inhibition kinetics towards HDACs depending on which complex they are incorporated in, researchers set out to identify HDACi with specificity towards CoREST for use in neurological diseases. They identified compound Rodin-A (Figure 4C), which showed a decrease in hematological toxicity compared with a non-selective HDACi. Rodin-A has a 139-fold selectivity towards CoREST compared with a 123-fold selectivity over Sin3 and NuRD and an 88-fold selectivity over NCoR [150]. An excellent example in which fundamental knowledge of protein interactions and structures aids rational drug design is given by the development of corin (Figure 4D), a synthetic dual inhibitor directed towards the two catalytically active sites of enzymes in the CoREST complex. The researchers combined the HDACi entinostat (Figure 2A) and an LSD1 inhibitor, a tranylcypromine analog, in a bi-valent molecule with a short linker that showed increased selectivity towards CoREST and prolonged inhibition kinetics due to irreversible binding of the LSD1 inhibitor [151]. This confirms the notion that molecules targeted towards multiple proteins in one complex increase selectivity towards that specific complex.

### 7.5. The SMRT/NCoR Complex

#### 7.5.1. Structure of SMRT/NCoR

SMRT and NCoR are co-repressor proteins with great homology that interact with nuclear hormone receptors, such as retinoid and thyroid hormone receptors, and with several other proteins to repress transcription [152,153]. SMRT forms complexes with HDAC3 and HDAC4, but only HDAC3 gets activated through a deacetylase-activating domain (DAD) that includes one of two SANT motifs located on the SMRT protein [154]. NCoR possesses a similar DAD domain determined by homology. In addition to HDAC3, SMRT and NCoR also form complexes with GPS2 and TBL1 that both interact with a conserved core region located on the SMRT/NCoR proteins named repression domain-1 (RD1) [152,155,156,157]. Furthermore, SMRT and NCoR recruit other HDACs such as HDAC5, HDAC7 and HDAC9 [158]. The exact role of these class IIa HDACs in this complex has not been determined yet, although HDAC3 is likely to be the sole enzymatic subunit responsible for deacetylase activity, since the other HDACs do not possess catalytic activity of their own [159,160]. Importantly, inhibition of HDAC3 should therefore fully abolish the deacetylase function of the SMRT/NCoR complex. Also, interaction with the Sin3 complex, and thus HDAC1 and HDAC2, has been described. However, it is anticipated that this interaction is not a core characteristic of SMRT/NCoR [161].

#### 7.5.2. Roles of SMRT/NCoR

The role of SMRT/NCoR is well defined. Characterization of NCoR-deficient mice showed its role in central nervous system development and in the development of T-lymphocytes and erythrocytes [162]. SMRT on the other hand fulfills a critical role in forebrain development and in the determination of neuronal stem cell fate [163]. Additionally, a fundamental role in the development of the heart is described [164]. In addition to its roles in development, SMRT/NCoR is involved in the regulation of the alternative activation pathway of macrophages. While classically LPS-activated macrophages are polarized to contribute to the progression of inflammation, alternatively IL-4-activated macrophages are polarized to secrete anti-inflammatory mediators. The HDAC3/SMRT/NCoR complex has been shown to suppress the IL-4-activated alternative pathway and is thereby believed to act as a brake for alternative activation [165]. Loss of HDAC3 removes this brake and thereby promotes the IL-4-induced alternatively activated phenotype [166]. Alternatively, the HDAC3/NCoR complex is proposed to suppress the transcription of the pro-inflammatory iNOS gene in classically activated macrophages [167]. In LPS-induced macrophages, this complex is degraded through the ubiquitin conjugating/19S proteasome system recruited by TBL1 [168], leading to upregulation of the iNOS expression. Conversely, HDAC3 is also reported to be required for the activation of inflammatory genes in classically activated macrophages, as evidenced from a HDAC3 knockout study [72]. This study, however, did not knock-out NCoR or SMRT, and the effect of the entire complex on inflammatory gene expression thereby remains to be investigated. Altogether, HDAC3/NCoR represses the expression of both pro- and anti-inflammatory genes in macrophages. In classically activated macrophages, the repression of pro-inflammatory genes is abolished by destruction of the complex. In alternatively activated macrophages the complex represses anti-inflammatory genes. We therefore predict that inhibition of HDAC3 is most beneficial in boosting the polarization of macrophages to become alternatively activated upon IL-4 stimulation.

#### 7.5.3. Targeting SMRT/NCoR

The described repression of pro-inflammatory genes by HDAC3/SMRT/NCoR in LPS-stimulated macrophages suggests that inhibition of HDAC3 might aid in lifting the repression and cause an increase in the expression of pro-inflammatory genes. However, several studies report that treatment of LPS-activated macrophages with the HDAC3 selective inhibitor RGFP966 does not have a significant impact on pro-inflammatory gene expression, except at relatively high concentrations, at which RGFP966 also inhibits HDAC1 and HDAC2 [81]. This could be a consequence of the described removal of the HDAC3/SMRT/NCoR complex from the chromatin upon LPS stimulation, thereby rendering inhibition useless. Yet, in the same study, siRNA mediated knock-down of HDAC3 yields a robust attenuation of LPS-induced inflammatory gene expression. It is, however, possible that without HDAC3, the rest of the SMRT/NCoR complex is not removed, perhaps by a failure to recruit TBL1 and the proteasomal degradation system. The remaining SMRT/NCoR complex could retain its repressing properties by mere occupation of inflammatory promotor sequences [169], while absence of unbound HDAC3 attenuates the inflammatory response. Alternatively, in IL-4-stimulated macrophages RGFP966 clearly enhances the expression of anti-inflammatory genes and promotes the alternative activation of macrophages. Future investigations should investigate whether the effects of either HDAC3 inhibition or knock-out in macrophages translates to a beneficial effect on inflammation in asthma or COPD.

### 7.6. The Role of Inositol Phosphates in HDAC Complex Formation

HDAC1/2/3 have an inositol phosphate (InsP) binding site and binding of InsP enhances the deacetylase activity of HDAC complexes. In a study done in embryonic stem cells, mutation of the inositol tetraphosphate binding site of HDAC1 and HDAC2 resulted in reduced HDAC activity in vivo [170]. A different study done in yeast also suggested a critical role for this allosteric site in regulation of HDAC activity [171]. The InsP binding site is a positively charged binding pocket that facilitates the binding of the multiple negatively charged phosphate groups of InsP. It is located close to the active site and allows for binding with co-repressor proteins. Co-repressor proteins and HDAC enzymes are simultaneously in contact with InsP and InsP can thus be viewed as a form of molecular glue, bonding the complex together [133,172,173]. A modeling study done on the crystal structure of HDAC3 in complex with SMRT/NCoR and InsP suggests that binding of InsP and DAD stabilize the backbone of HDAC3 [174]. Also, the HDAC activity can increase or decrease depending on the type of InsP that binds. Allosteric inhibition of HDAC1/2/3 by InsP mimics may be possible, but the generic role of InsP in many different signaling pathways would greatly complicate such an approach.

## 8. Future Perspectives

We have summarized the beneficial effects of both selective and more general HDACi on the inflammatory mechanisms of both asthma and COPD that require further investigation. One area of further research is to combine the features of isoform selective inhibitors to yield unique selectivity profiles that could potentially further improve their positive benefit-risk ratios. A uniquely selective HDAC3/6 inhibitor, for instance, would combine the attenuation of cytokine expression by inhibition of HDAC3 with the reduction of immune cell motility by inhibition of HDAC6, without eliciting the pleiotropic effects of HDAC1/2 inhibition. For instance, an HDAC3/6 inhibitor of unknown structure reproduced the anti-inflammatory effects of reduced expression of HDAC3 in an in vitro model of rheumatoid arthritis [175]. Other unique combinations left to explore are HDAC1/2/6, HDAC1/2/8 and HDAC3/8 inhibitors. A dual action HDAC6/8 inhibitor already exists, but has not been tested yet in inflammatory models [176].

Furthermore, the finding that HDACs exist in specific complexes has been used to design inhibitors for specific complexes, like the dual-warhead LSD-1/HDAC1/2/3 inhibitor corin (Figure 4D) directed to CoREST. The availability of structural information of the specific HDAC complexes will aid the discovery of comparable examples. Besides, further modification of *o*-aminoanilide-type HDACi might similarly yield complex-selective inhibitors as exemplified by Rodin-A (Figure 4B).

**Figure 5:**
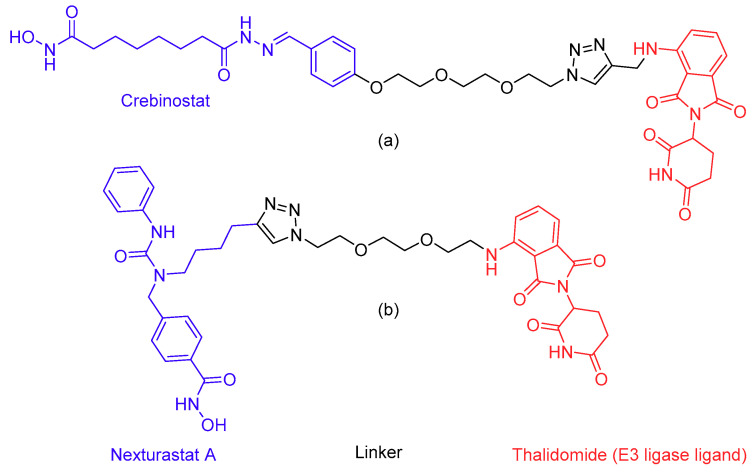
Structures of histone deacetylase 6 selective proteolysis targeting chimeras. A histone deacetylase inhibitor is connected to the E3 ligase ligand thalidomide with a hydrophilic linker. (**a**) Compound **9**c, which contains the histone deacetylase inhibitor crebinostat. (**b**) Compound NP8, which contains the histone deacetylase inhibitor nexturastat A.

Lastly, due to the important structural role of HDACs in protein complexes, prevention of HDAC complex formation by protein–protein interaction inhibitors is an interesting option. A more novel but related approach is to target enzymes for degradation using the proteolysis targeting chimera (PROTAC) technique [177]. In this respect, PROTAC molecules for the selective degradation of HDAC6 (Figure 5) have recently been developed [178,179]. The compounds consist of an HDAC inhibitor directed to the HDAC catalytic site and the E3 targeting ligand thalidomide, to bind to an E3 ubiquitin ligase complex, coupled together with a linker [180,181,182]. It is interesting to note that while compound **9**c (Figure 5A) contains the non-selective inhibitor crebinostat [183], it is reported to selectively degrade HDAC6. This may be the result of the formation of a stable ternary complex between the E3 ligase and HDAC6 and not with the other HDACs [182], but further research is needed to confirm this. Compound Np8 (Figure 5B) contains the HDAC6 selective inhibitor nexturastat A [184]. Finally, small molecules that selectively degrade HDAC1/2 or 3 have not yet been developed, and this is therefore an interesting area for further research. PROTACs for specific complexes may even be developed by adding ubiquitin ligase functionalities to the described complex-specific inhibitors. With these new strategies, the validation of HDACs as targets for improved therapies for asthma and COPD enters an exciting new era.

## Figures and Tables

**Table 1:** Effects of histone deacetylase inhibitors in in vivo models of asthma and COPD. The plus sign (+) indicates inhibition and the minus sign (−) indicates no inhibition of the respective histone deacetylase isoforms at relevant concentrations.

In Vivo Model		Histone Deacetylase						Effect
		Inhibitor	1	2	3	6	8	
Asthma	Chronic asthmatic mouse model [84]	Tubastatin A	−	−	−	+	−	Reduced inflammation
		PCI-34051	−	−	−	−	+	Reduced hyperresponsiveness and inflammation
	Murine innate allergic lung inflammation [90]	Trichostatin A	+	+	+	+	+	Decreased amount of inflammatory cells and inflammatory proteins
Chronic Obstructive Pulmonary Disease	Cigarette smoke exposed mice [82]	Entinostat	+	+	+	−	−	Reduced expression of IL-8 and decreased influx of neutrophils
	Cigarette smoke exposed mice [89]	Tubastatin A	−	−	−	+	−	Protection from cigarette-smoke induced mucociliary clearance disruption
